# Earlier reperfusion in patients with ST-elevation Myocardial infarction by use of helicopter

**DOI:** 10.1186/1757-7241-20-70

**Published:** 2012-10-04

**Authors:** Lars Knudsen, Carsten Stengaard, Troels Martin Hansen, Jens Flensted Lassen, Christian Juhl Terkelsen

**Affiliations:** 1 Helicopter Emergency Medical Service, Central Denmark Region, Aarhus, Denmark; 2 Department of Prehospital Medical Services, Central Denmark Region, Aarhus, Denmark; 3 Department of anesthesiology, Aarhus University Hospital, Nørrebrogade, 8000, Aarhus, Denmark; 4 Department of cardiology B, Aarhus University Hospital in Skejby, Aarhus, Denmark

**Keywords:** STEMI, Angioplasty, HEMS, Helicopter, Field-triage, System delay

## Abstract

**Background:**

In patients with ST-elevation myocardial infarction (STEMI) reperfusion therapy should be initiated as soon as possible. This study evaluated whether use of a helicopter for transportation of patients is associated with earlier initiation of reperfusion therapy.

**Material and methods:**

A prospective study was conducted, including patients with STEMI and symptom duration less than 12 hours, who had primary percutaneous coronary intervention (PPCI) performed at Aarhus University Hospital in Skejby. Patients with a health care system delay (time from emergency call to first coronary intervention) of more than 360 minutes were excluded. The study period ran from 1.1.2011 until 31.12.2011. A Western Denmark Helicopter Emergency Medical Service (HEMS) project was initiated 1.6.2011 for transportation of patients with time-critical illnesses, including STEMI.

**Results:**

The study population comprised 398 patients, of whom 376 were transported by ambulance Emergency Medical Service (EMS) and 22 by HEMS. Field-triage directly to the PCI-center was used in 338 of patients. The median system delay was 94 minutes among those field-triaged, and 168 minutes among those initially admitted to a local hospital. Patients transported by EMS and field-triaged were stratified into four groups according to transport distance from the scene of event to the PCI-center: ≤25 km., 26–50 km., 51–75 km. and > 75 km. For these groups, the median system delay was 78, 89, 99, and 141 minutes. Among patients transported by HEMS and field-triaged the estimated median transport distance by ground transportation was 115 km, and the observed system delay was 107 minutes. Based on second order polynomial regression, it was estimated that patients with a transport distance of >60 km to the PCI-center may benefit from helicopter transportation, and that transportation by helicopter is associated with a system delay of less than 120 minutes even at a transport distance up to 150 km.

**Conclusion:**

The present study indicates that use of a helicopter should be considered for field-triage of patients with STEMI to the PCI-center in case of long transportation. Such a strategy may ensure that patients living up to 150 km. from the PCI-center can be treated within 120 minutes of emergency call.

## Introduction

Ischemic heart disease is the leading cause of death in the western world. In patients with ST-elevation Myocardial Infarction (STEMI) reperfusion with either fibrinolysis or primary percutaneous coronary intervention (PPCI) is associated with improved outcome. Reperfusion with PPCI is superior to fibrinolysis, when the extra time necessary to perform PPCI compared to fibrinolysis (PCI-related delay) is less than 120 minutes [1-3]. In Denmark, PPCI is recommended as a national reperfusion strategy [4]. However, in rural areas it may be a challenge to ensure a PCI-related delay below 120 minutes, when using a traditional ambulance Emergency Medical Service (EMS) [1,5]. A 24–7 Helicopter Emergency Medical Service (HEMS) was introduced 1.6.2011 in the Western Part of Denmark. The HEMS was located at the airport in Karup, at a distance of 70 km. from the single PCI-center (Aarhus University Hospital in Skejby) in the Central Denmark Region. The HEMS provides prehospital care for patients with time-critical illness or injury, including STEMI. The HEMS service was organized similarly to HEMS systems in Scandinavia and central Europe with a HEMS crew comprised of an experienced prehospital trained anesthetist, a pilot, and a paramedic. An acute medical coordinating center (AMK) was responsible for dispatch of both the EMS and HEMS. The purposes of the present study were: 1) to evaluate whether use of HEMS for transportation of patients with STEMI was associated with earlier initiation of reperfusion therapy when compared with patients transported by ground transportation, and 2) to estimate the distance from the scene of event to the PCI-center at which a reduction in health care system delay (defined as time from emergency call to first coronary intervention) is expected when using helicopters for transportation instead of ground transportation.

## Methods and study population

A prospective study was conducted in the Central Denmark Region, covering an area of 13.053 km2, and with 1.237.041 inhabitants. The study period ran from January 1^st^ 2011 to December 31^st^ 2011. The study population comprised patients with STEMI and symptom duration of 12 hours or less, who had PPCI performed at Aarhus University Hospital in Skejby. Also patients with return of spontaneous circulation after cardiac arrest, who had a prehospital or local hospital ECG with signs of STEMI, were included. Prehospital ECGs were transmitted to a telemedicine center in Skejby, Herning, Viborg or Horsens, and in case of STEMI field-triage to the PCI-center in Skejby was recommended. Prehospital data were prospectively registered at the AMK-center responsible for dispatching both the EMS and the HEMS.

A structured criteria-based dispatching tool (Danish index for Emergency Medical Dispatch) was used for dispatch of EMS and HEMS [6]. Local ambulances and emergency physicians were dispatched in suspected AMI-cases, and HEMS in addition when the estimated transportation time to the PCI-center was longer than 30 minutes. In cases where EMS arrived before the helicopter, and the ECG transmitted to a telemedicine center was without signs of STEMI, and the patient was stable, HEMS was cancelled, and returned to base. Otherwise HEMS landed as close to the scene of event as possible (>92% within 500 m from the patient). All patients triaged to PPCI were treated with aspirin, clopidogrel, iv. heparin and opoid as analgesic. On-site times for the HEMS were < 10 minutes in uncomplicated cases. Planning and optimization of the arrival of the patient at the PCI-center was started immediately after HEMS take-of. Communicating arrival time and patient data to the PCI-center allowed catheterization laboratory personal to prepare for the procedure. Landing site for the HEMS right next to the emergency entrance and direct patient transfer on a stretcher from the helicopter to the PCI-lab allowed quick transfer from landing to arrival in the PCI-lab.

Data on the invasive procedure as well as baseline characteristics were prospectively registered in the Western Denmark Heart Registry. Patients self-presenting at the local hospital or at the PCI-center, and patients with a system delay of more than 6 hours were excluded from the analyses. Patients were stratified according to the estimated transport distance from scene of event to the PCI-center if ground transportation by EMS had been used, and according to mode of transportation (EMS or HEMS). The estimated transport distance was calculated using a web-based service (http://www.krak.dk), entering the address of the scene of event and the PCI-center. The study did not require approval from the Ethical Committee. Approval from the national data protection agency and the national board of health was granted.

### Statistics

Categorical variables are presented as n (proportion or per centage) and continuous variables as median (IQR). Chi-square test was used for comparison of categorical variables and Kruskall Wallis test for comparison of continuous variables. A second order polynomial regression analysis (quadratic) was performed to evaluate the association between transportation distance and system delay in patients transported by EMS and HEMS, respectively, and quadratic plots presented. The intersection between the two regression lines were interpreted as the distance from the PCI-center beyond which helicopter transportation is expected to be associated with earlier initiation of reperfusion therapy. CJT performed the statistical analyses using STATA 12.

## Results

**Figure 1:**
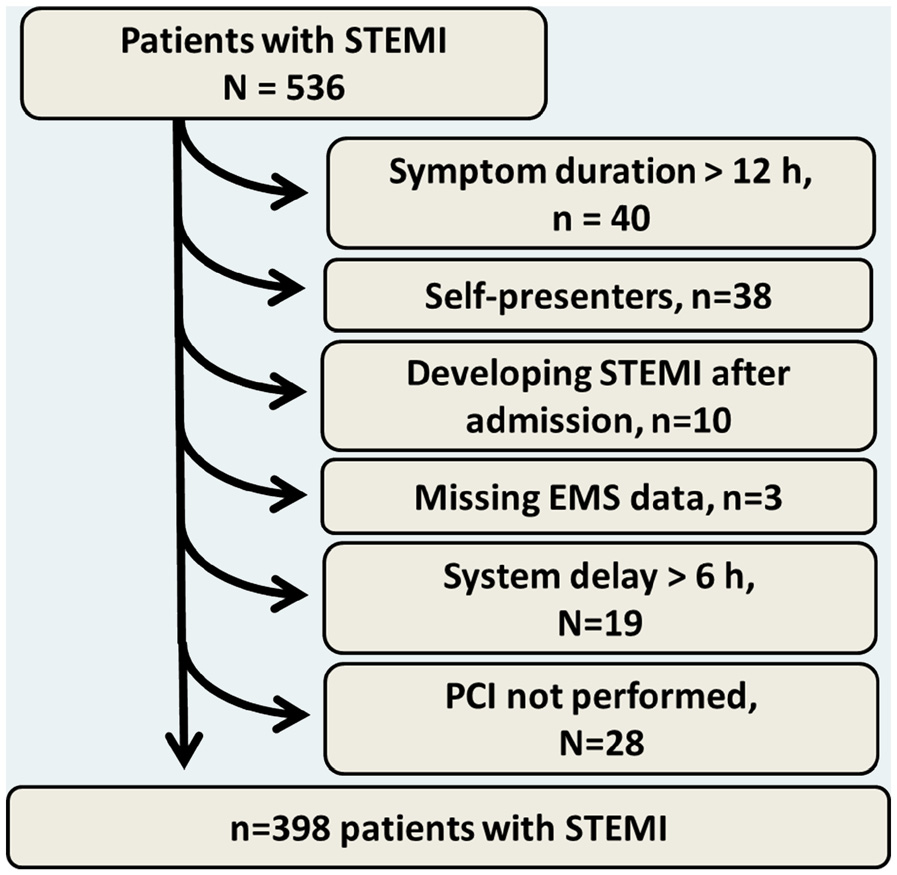
**Selection of patients.** EMS = Emergency Medical Service. PCI = Percutaneous coronary intervention. STEMI = ST-Elevation Myocardial Infarction. System delay = Delay (minutes) from emergency call to first coronary intervention.

**Figure 2:**
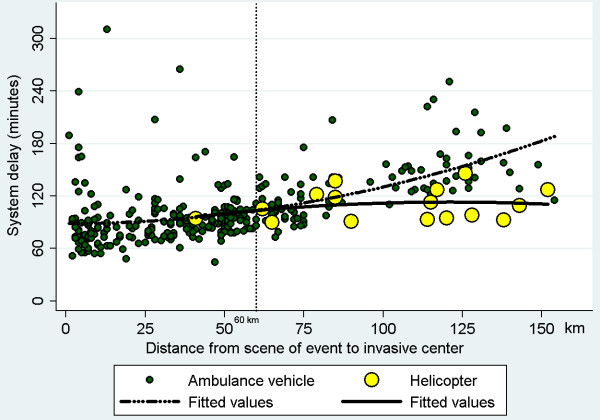
**Association between health care system delay (time from emergency call to first coronary intervention) and distance from the scene of event to the Percutaneous Coronary Intervention center in patients with ST-elevation myocardial infarction.** Stratified according to mode of transportation (by ambulance vehicle or helicopter). Plots based on polynomial regression analysis describes the association between transport distance and system delay in patients transported by ambulance and helicopter, respectively.

During the study period 536 patients with STEMI were admitted for PPCI at Aarhus University Hospital in Skejby. Symptom duration was more than 12 hours in 40 patients, 38 patients were self-presenters, 10 patients developed STEMI after admission to one of 6 hospitals in the region (Herning, Viborg, Silkeborg, Randers, Horsens, Aarhus), and in 3 patients it was uncertain whether they were transported from the scene of event by EMS. In 19 patients system delay was above 6 hours, and in 28 patients PPCI was not performed. The study population thus comprised 398 patients with STEMI (Figure 1). Among 376 (94%) patients transported by ambulance the distance from the scene of event to the PCI-center was ≤25 km. in 98 patients (Group A), 26–50 km. in 102 patients (Group B), 51–75 km. in 101 patients (Group C) and >75 km. in 75 patients (Group D). Among 22 (6%) patients transported by helicopter the median distance from the scene of event to the PCI-center was 104 km (Group E). Baseline characteristics for the 5 group of patients are shown in Table 1. The median system delay was 78,89,99,141 and 107 minutes in the five group of patients if field-triaged, and 161,188,192,160 and 154 minutes among patients not-field-triaged. When only focusing on patients field-triaged, and using second order polynomial regression analysis, the association between transportation distance (x) to the PCI-center and system delay (Y) was: Y = 88.60-0.0020*x + 0.0042*x^2^ in patients transported by EMS, and Y = 74.8 + 0.632*x-0.00261*x^2^ in patients transported by the HEMS. The two regression lines intersected at a transportation distance of 60 km. The estimated reduction in system delay when using helicopters instead of ground transportation to field-triage patients was 18, 31, 60 and 72 minutes at a transport distance of 100, 115, 140 and 150 km from the PCI-center (Figure 2).

**Table 1:** Baseline characteristics according to the mode of transportation (ambulance vehicle or helicopter) and according to the distance from the scene of event to the PCI-center (Hospital with primary percutaneous coronary intervention facilities)

	**Group A (0–25 km.) n = 98**	**Group B (26–50 km.) n = 102**	**Group C (51–75 km.) n = 101**	**Group D (>75 km.) n = 75**	**Group E n = 22**	**All patients N = 398**
**Field-triage to PCI-center,%**	89% (87/98)	93% (95/102)	83% (84/101)	75% (56/75)	73% (16/22)	85% (338/398)
**Distance, scene of event to PCI-center, km †**	8 (5–14)	39 (33–47)	61 (53–67)	114 (87–123)	104 (79–128)‡	50 (26–74)
**- Distance among patients field-triaged**	8 (5–13)	38 (32–47)	60 (53–67)	115 (102–127)	115 (82–127)	49 (23–69)
**- Distance among patients not field-triaged**	12 (7–20)	41 (39–47)	67 (56–68)	92 (86–122)	90 (62–129)	67 (42–88)
**Health Care System delay, all patients, minutes**	82 (70–104)	90 (80–104)	104 (92–122)	141 (125–166)	116 (94–137)	100 (85–134)
**- System delay among patients field-triaged**	78 (68–93)	89 (79–100)	99 (91–111)	141 (126–156)	107 (94–124)	94 (82–119)
**- System delay among patients not field-triaged**	161 (141–200)	188 (132–248)	192 (142–220)	160 (120–199)	154 (125–165)	168 (136–216)
**Door-to-balloon delay, minutes**	34 (24–49)	28 (22–37)	26 (23–38)	31 (23–46)	37 (24–60)	29 (23–43)

†: If transported by ambulance vehicle. ‡: One patient living on an island was not included because distance by ground transportation could not be calculated. Door-to-balloon delay: Delay form arrival at the PCI-center to first coronary intervention. PCI: Percutaneous coronary intervention. Health Care System delay: Delay from Emergency Call to first coronary intervention. Continuous data presented as median (IQR) and categorical data presented as percentage (number/valid cases).

## Discussion

The present study describes the initial experience using helicopter for transportation of patients with STEMI in the Central Denmark Region, and the overall finding is that use of helicopter enables reperfusion therapy within 120 minutes of emergency call in patents living up to 150 km. from the PCI-center. This is the first study to address system delay in patients with STEMI transported by HEMS. The study shows that patients transported by helicopter had a median transport distance of 104 km and a median system delay of 116 minutes, i.e. shorter delay compared to patients transported by EMS with a transport distance to the PCI-center of more than 75 km. It was estimated that there was an expected reduction in system delay if using helicopter for transportation instead of ground transportation when the distance to the PCI-center by ground transportation was above 60 km. Among patients living 150 km. from the PCI-center the estimated reduction in system delay was more than one hour when using a helicopter for transportation. Moreover, use of a helicopter for transportation seemed to be associated with a system delay of less than 120 minutes even at a distance up to 150 km. from the PCI-center. There is a potential for improvement in logistics, as only 73% of patients transported by helicopter were field-triaged directly to the PCI-center for PPCI, and as helicopters were only used to transport a minority of patients with a transportation distance to the PCI-center above 60 km. It is expected that the proportion of patients eligible for helicopter transportation will increase if the helicopter is dispatched routinely in case of heart attacks. Larger studies are needed to confirm the present findings. Furthermore, the distance from the PCI-center at which use of helicopters may be associated with earlier initiation of therapy is of course dependent on infrastructure, and will vary between regions and countries. It is expected that helicopters may be beneficial in other time-critical illnesses including cardiogenic shock, malignant arrhythmia, out of hospital cardiac arrest, cerebral stroke and of course trauma cases. Based on the present findings it is estimated that patients living 115 km. from the PCI-center will be treated approximately 30 minutes earlier and patients living 140 km. from the PCI-center will be treated one hour earlier if transported by HEMS instead of EMS. Previous studies have shown that a one-hour earlier initiation of reperfusion therapy is associated with a 10% relative reduction in mortality, and a 10% reduction in the risk of developing congestive heart failure following STEMI [7,8]. The extra delay acceptable to perform PPCI instead of administering fibrinolysis is around 120 minutes based on previous metaanalyses of randomized controlled trials, and based on recent registry studies [1,2]. Given the fact that 30–45 minutes elapse from Emergency Call to fibrinolysis even in the case of prehospital fibrinolysis [9], one reperfusion strategy with PPCI for all seems to be the ideal strategy in Denmark. In other countries with longer transportation time to PCI-centers, prehospital fibrinolysis or a pharmaco-invasive approach with prehospital fibrinolysis combined with transfer for acute angiography may prove beneficial. The ongoing STREAM trial will further clarify the role of a pharmacoinvasive strategy.

### Limitations

This was a descriptive study, and any findings should be interpreted with caution. The study population was limited in size, and larger studies are needed to confirm the findings and more accurately determine the distance from the PCI-center at which transportation with HEMS instead of EMS is associated with earlier initiation of reperfusion therapy. The primary strength of this study is that an unselected cohort of patients with STEMI was identified, who were transported from the scene of event to a PCI-center by either traditional EMS or HEMS.

## Conclusion

The present study indicates that use of a helicopter should be considered for field-triage of patients with STEMI to the PCI-center in case of long transportation. Such a strategy may ensure that patients living up to 150 km. from the PCI-center can be treated within 120 minutes of emergency call.

## Competing interests

LK is head of the helicopter emergency medical service in the central region of Denmark.

## Authors' contributions

The study concept and design was conceived by LK, CJT, JFL. Acquisition of data was done by CJT, LK, CS, TMH. Interpretation of data and drafting of manuscript was done by CJT. Critically review and acceptance of the manuscript was done by CJT, LK, CS, TMH and JFL. All authors approved the final version to be submitted.
